# Supplementary material for: Patient-derived xenograft culture-transplant system for investigation of human breast cancer metastasis
Source: Commun Biol. 2021 Nov 5;4:1268. doi: 10.1038/s42003-021-02596-y (PMC8571269; doi:10.1038/s42003-021-02596-y)
Supplement: Supplementary file 2 — Supplementary Information [file 42003_2021_2596_MOESM2_ESM.pdf]

Supplementary Information: Supplementary Figures

Patient-derived xenograft culture-transplant system for investigation of human breast cancer metastasis

Dennis Ma[1†], Grace A. Hernandez[2†], Austin E.Y.T. Lefebvre[3], Hamad Alshetaiwi[2,4], Kerrigan Blake[5], Kushal R. Dave[2], Maha Rauf[1], Justice W. Williams[1], Ryan T. Davis[2], Katrina T. Evans[2], Aaron Longworth[2], Madona Y.G. Masoud[2], Regis Lee[2], Robert A. Edwards[6], Michelle A. Digman[3], Kai Kessenbrock[1], and Devon A. Lawson[2*]

[1]Department of Biological Chemistry, University of California, Irvine, Irvine, CA, USA, 92617

[2]Department of Physiology and Biophysics, University of California, Irvine, Irvine, CA, USA, 92617

[3]Department of Biomedical Engineering, University of California, Irvine, Irvine, CA, USA, 92617

[4]Department of Pathology, University of Hail, Hail, 2440, Saudi Arabia

[5]Center for Complex Biological Systems, University of California, Irvine, Irvine, CA, USA, 92697

[6]Department of Pathology & Laboratory Medicine, University of California, Irvine, CA, USA, 92617

[†]These authors contributed equally
*Address correspondence to

Devon A. Lawson, Ph.D.
Department of Physiology and Biophysics
University of California, Irvine
839 Health Sciences Road, Sprague Hall 112
Irvine, CA 92617-3905
Tel: 949-824-4113
email: dalawson@uci.edu

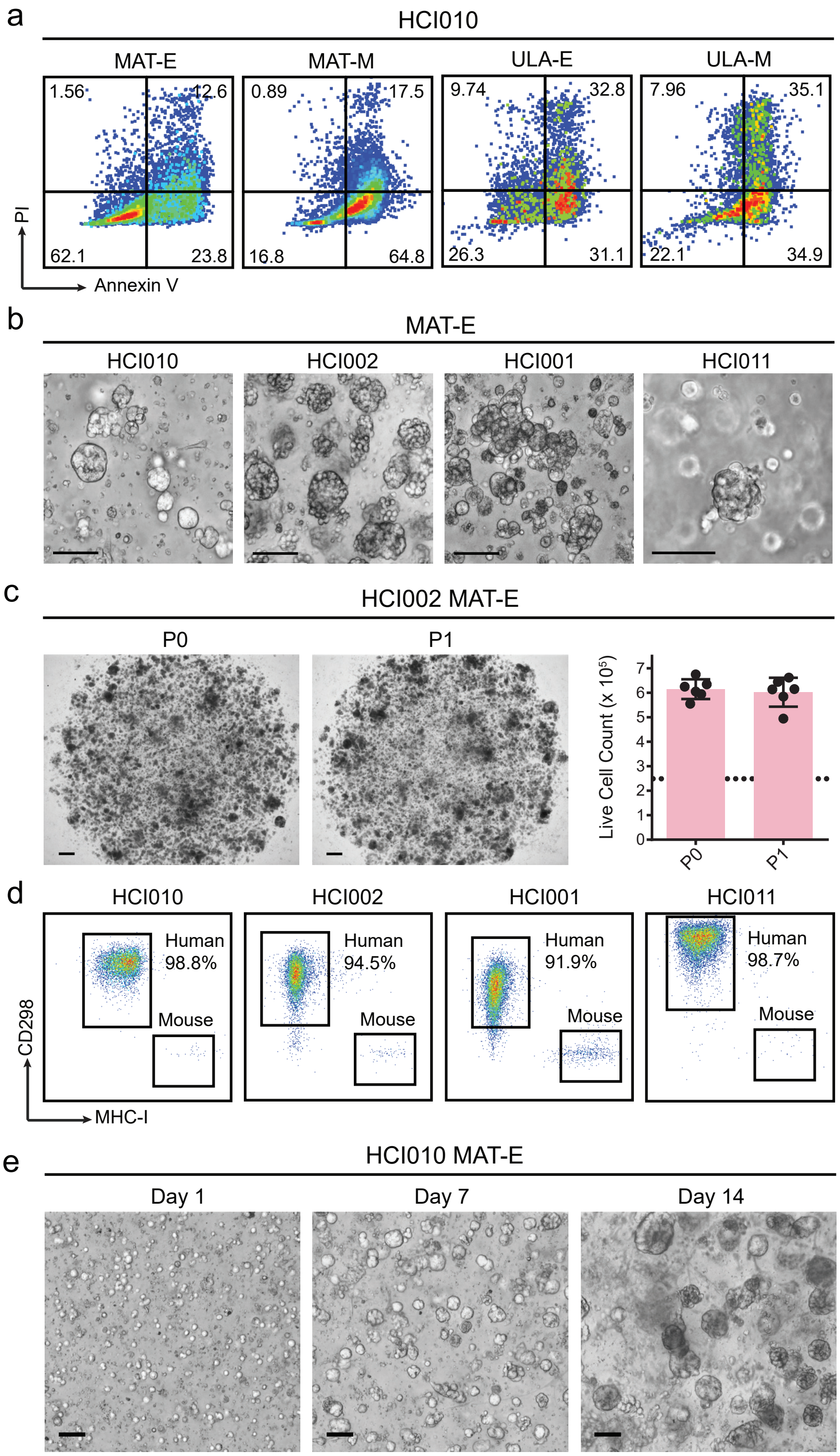

Supplementary Figure 1: Comparison of PDX cell culture conditions. (a) Representative flow cytometry plots show viability of HCI010 cells by aV and PI staining after culture as described in Fig 1a. **(b)** Representative brightfield images show spheres generated by HCI010, HCI002, HCI001 and HCI011 tumor cells grown in MAT-E culture conditions. Scale bar= 100 μm. **(c)** Analysis of cell number and viability in HCI002 passaging experiments. P0 and P1 cells were plated at 2.5 x 10^5 cells/well (dashed line) in MAT-E conditions and quantified nine days later by trypan blue exclusion. Brightfield images (left panels) show representative wells containing spheroid structures at day nine. Scale bars = 400 μm. Bar graph (right panel) shows total viable HCI002 cell number counted at day nine in P0 and P1 (n=6 wells each). Data represented as mean ± s.d. **(d)** Flow cytometry analysis to determine species identity of spheres generated in (b). Representative plots show the percent of human $CD298^+MHCI^-$ tumor cells in each culture. **(e)** Time-lapse imaging of sphere growth from HCI010 cells in MAT-E conditions. Representative brightfield images at day 1, 7, and 14 are shown. See also Supplementary videos 1-3. Scale bar= 100 μm.

a

PDX model	Cell number	Fraction of mice with tumors
HCI002 MAT-E	5.0×10^3	8/9
HCI002 MAT-E	1.0×10^4	10/10
HCI002 MAT-E	1.0×10^5	12/12

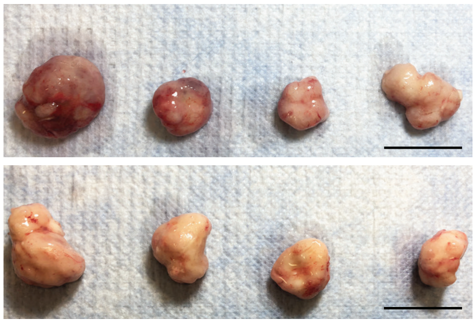

c

Metastatic site	HCI010 MAT-E	HCI010 [26]
Peripheral blood	2/5 (40.0%)	3/22 (13.6%)
Lymph node	5/14 (35.7%)	17/33 (51.5%)
Lung	11/14 (78.8%)	31/33 (93.9%)
Bone marrow	2/5 (40.0%)	6/23 (26.1%)
Brain	0/5 (0.0%)	1/13 (7.7%)

d

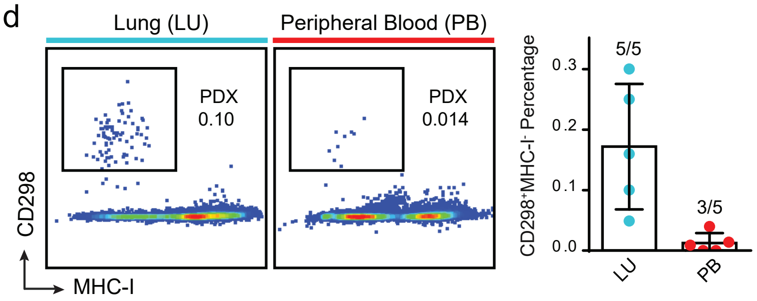

b

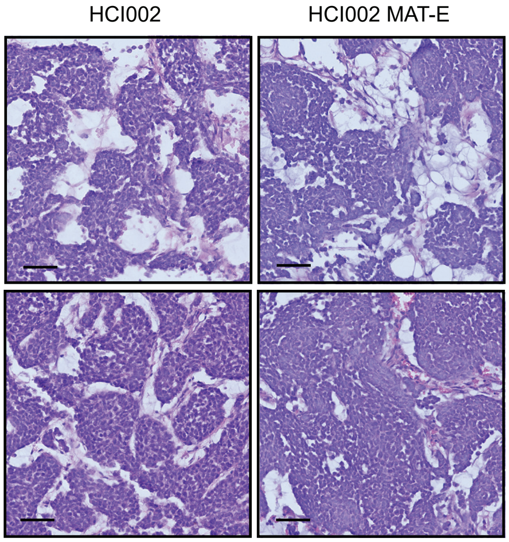

e

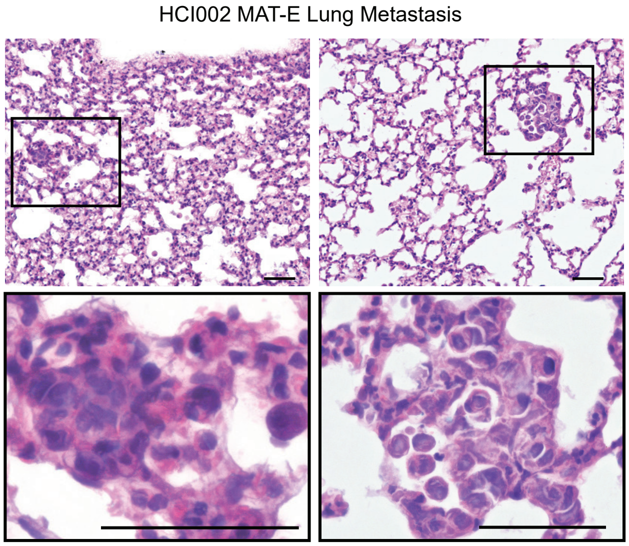

Supplementary Figure 2: Analysis of primary tumors and spontaneous metastases generated by cultured PDX cells. (a) Serial dilution and transplantation analysis to determine tumorigenic capacity of cultured cells. HCI002 cells were cultured in MAT-E conditions and injected orthotopically into NOD/SCID mice at increasing dilution (5.0×10^3 – 1.0×10^5). Table (top) shows the fraction of tumors generated at each dilution. Representative images (bottom) show primary tumors generated from orthotopic transplantation of 1×10^5 cells after 12 weeks *in vivo*. Scale bar = 1cm. **(b)** Histopathological analysis of tumors generated from uncultured (HCI002) and cultured (HCI002 MAT-E) cells. Representative images show tumor sections stained with hematoxylin and eosin (H&E). Scale bar = 50 µm. **(c)** Table shows the percentage of mice positive for metastasis in indicated tissues following orthotopic transplantation of cultured (HCI010 MAT-E) vs uncultured (HCI010) cells. Data for uncultured cells is taken from Lawson et al., 2015.[26] **(d)** Quantification of spontaneous metastasis in animals transplanted with 1×10^5 cultured HCI002 cells. Representative plots (left) show $CD298^+MHC\text{-}I^-$ human metastatic PDX cells in the lung and peripheral blood by flow cytometry. Bar graph (right) shows quantification of percentage of metastatic cells in a cohort of transplanted animals (n=5). Fractions indicate the number of tissues with metastasis, defined by >0.005% $CD298^+MHC\text{-}I^-$ cells. Data is represented as the mean ± s.d. LU = Lung, PB = peripheral blood. **(e)** Representative images of metastatic lesions in the lungs of animals transplanted with cultured HCI002 cells identified by H&E staining and histopathological analysis. Scale bar= 50 µm.

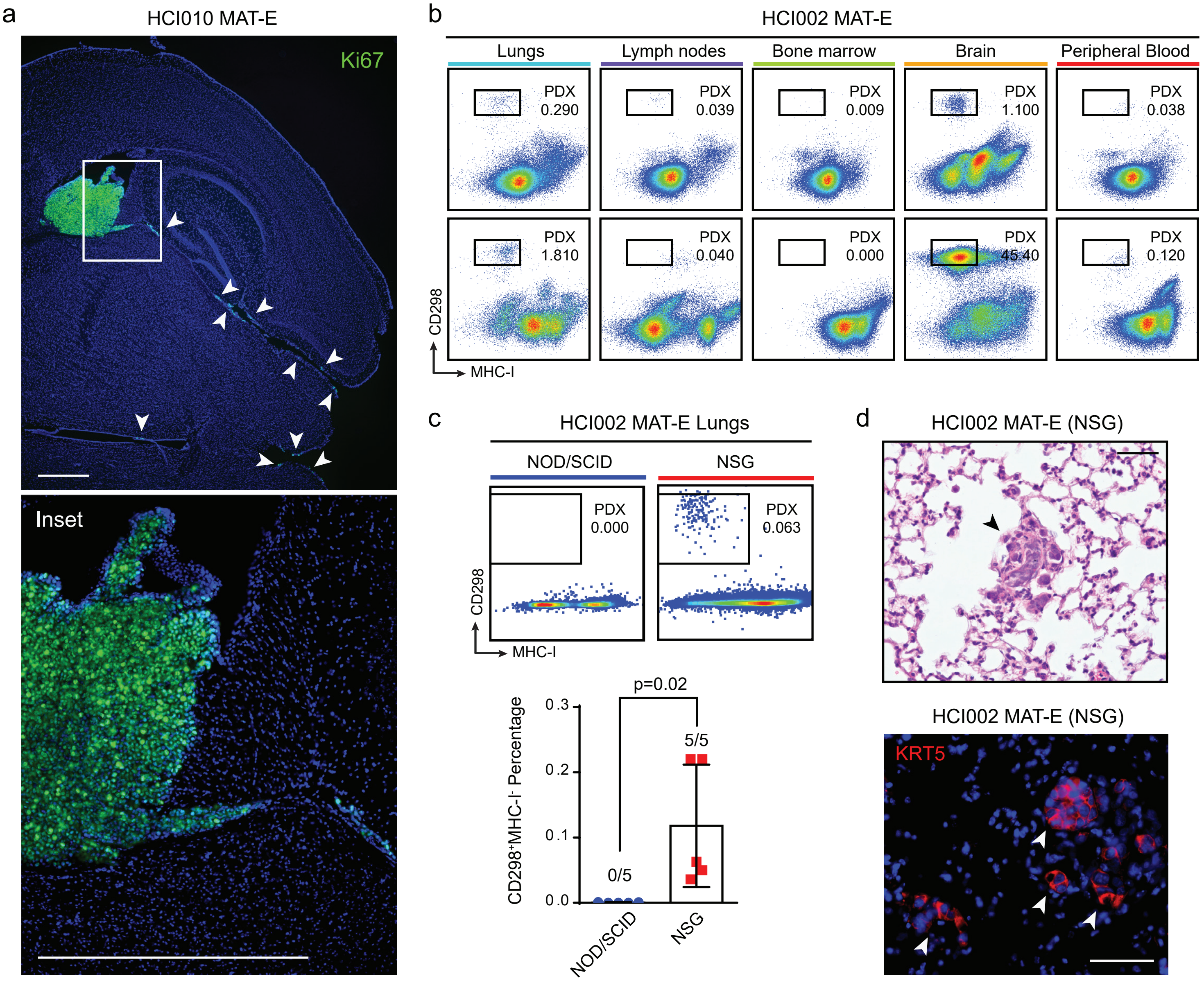

Supplementary Figure 3: Characterization of experimental metastasis produced by PDX sphere cells. (a) IF staining for Ki67 in brain metastatic lesion from Fig 5d. Inset shows higher power visualization of Ki67+ nuclei in 40 μm brain section. Arrows indicate micrometastatic lesions. Scale bar = 800 μm. **(b)** Analysis of metastatic spread following i.c. injection of cultured HCI002 cells *in vivo.* Representative flow cytometry plots show $CD298^{+}MHC\text{-}I^{-}$ human metastatic cells in the lungs, lymph nodes, bone marrow, brain, and blood 8 weeks following i.c. injection of 5×10^{5} cultured HCI002 cells into NOD/SCID mice. **(c)** Comparison of experimental metastasis from cultured HCI002 cells in NOD/SCID and NSG mice. Flow cytometry plots show percent of human $CD298^{+}MHC\text{-}I^{-}$ metastatic cells in the lungs of representative mice 8 weeks following i.v. injection of 5×10^{5} HCI002 cultured cells (top plots). Bar graph shows quantification of percentage of metastatic cells in a cohort of transplanted animals (n=5) (bottom). Fractions indicate the number of lungs with metastasis, as defined by >0.005% $CD298^{+}MHC\text{-}I^{-}$ cells. Data is represented as the mean ± s.d.. *P*-value was determined by unpaired t-test. **(d)** Images show representative metastatic lesions (arrows) in the lungs of NSG mice identified by H&E staining (top) and IF staining for KRT5 (bottom). Scale bar = 50 μm.

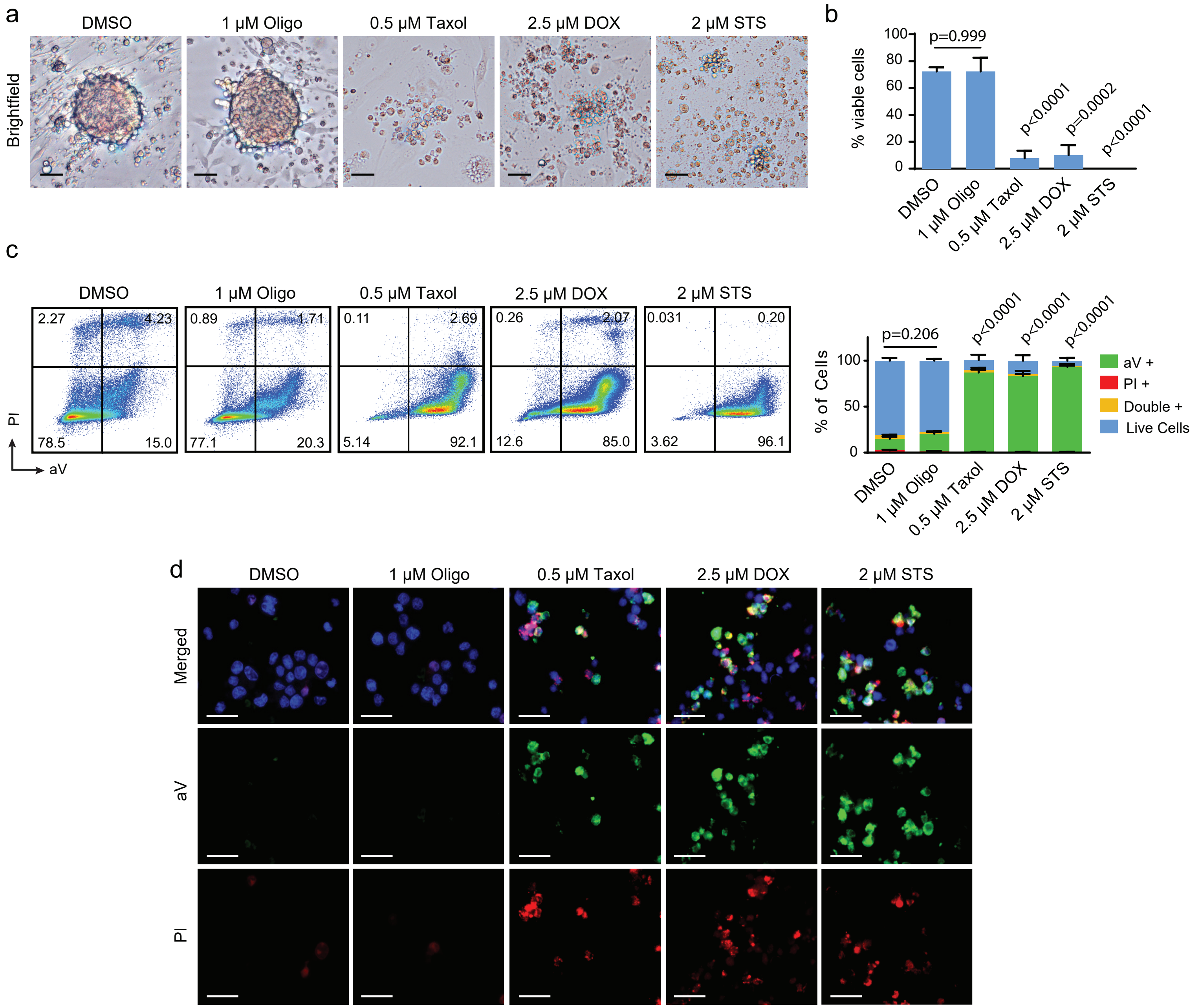

Supplementary Figure 4: Cytotoxicity and cell death analysis of PDX cells post drug treatment. (a) Z-stack brightfield images of representative HCI002 MAT-E spheroids grown for two weeks and treated with the indicated drugs for 72 hours. Scale bar= 50 µm. Oligo= oligomycin; DOX= doxorubicin; STS= staurosporine. **(b)** Trypan blue exclusion viability assay of HCI002 MAT-E cells grown for two weeks and treated with the indicated drugs for 72 hours. Values expressed as mean ± s.d. from triplicate wells (n=3). *P*-values were determined by unpaired t-tests of each condition vs. the DMSO control group. Taxol, DOX and STS were used as positive controls for cell death. **(c)** Representative flow cytometry plots of aV and PI dead cell analysis of HCI002 MAT-E cells treated for 72 hours. Bar graph shows percent of live and dead HCI002 cells by aV and PI. Data represented as mean ± s.d. (n=3). *P*-values were determined by unpaired t-tests of each condition vs. the DMSO control group for the percent of aV^-PI^- viable cells. Taxol, DOX and STS were used as positive controls for cell death. **(d)** Representative aV and PI fluorescence micrographs of HCI002 MAT-E cells treated for 72 hours. Blue= Hoechst nuclear counterstain. Scale bar= 25 µm.

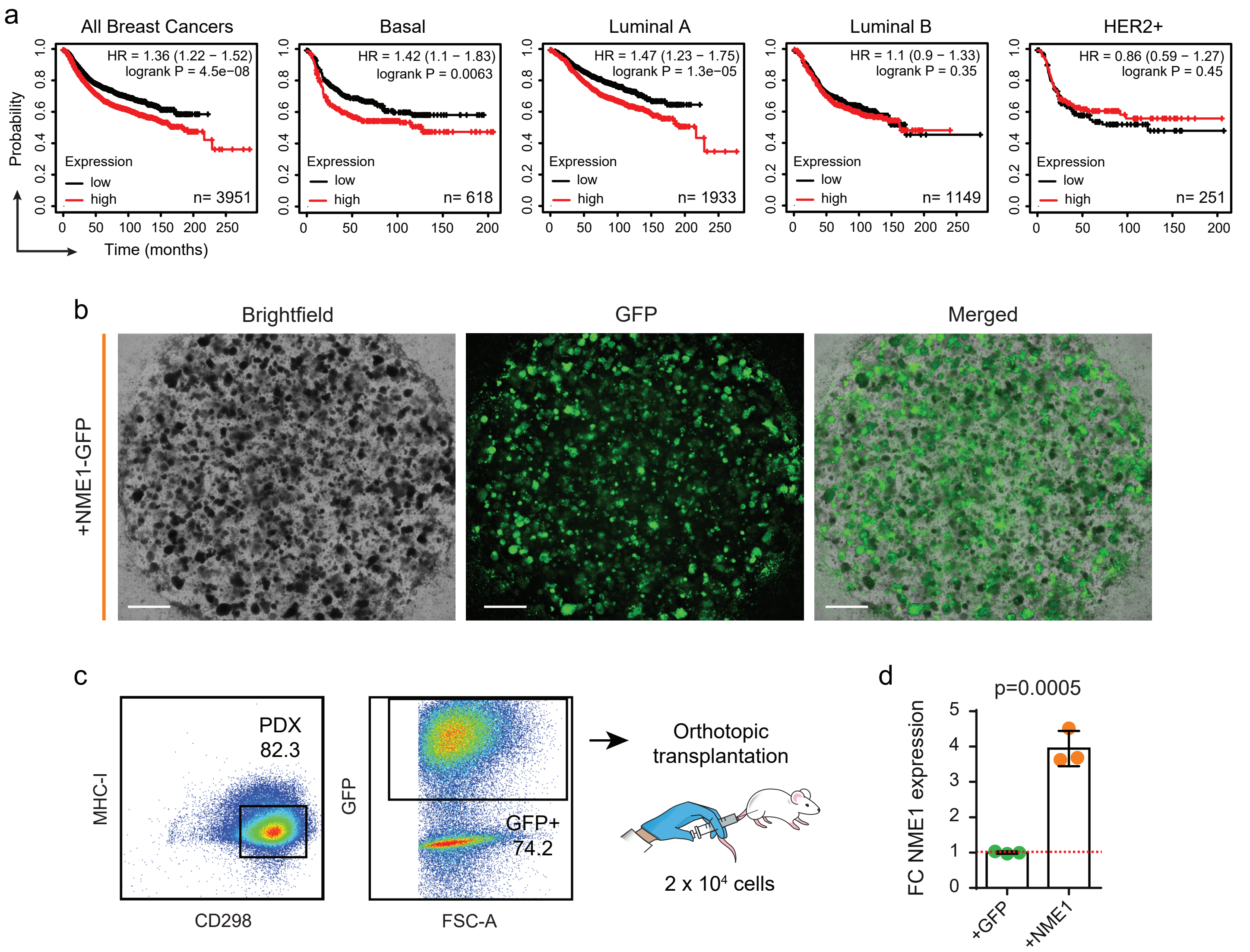

Supplementary Figure 5: NME1 overexpression in cultured PDX cells and its relevance in patient breast cancer prognosis. (a) Kaplan-Meier (KM) curves show relapse free survival (RFS) in breast cancer patients by tumor subtype using the KM plotter database[56], based on their primary tumor expression of NME1. *P*-values were determined via a log-rank test. **(b)** Images show GFP expression in PDX spheres cultures following lentiviral transduction *in vitro.* HCI010 sphere cells were transduced with +NME1-GFP lentivirus at MOI=25 and re-seeded in MAT-E culture at a density of 2 x 10^5 cells per well. Images show z-stack micrographs of individual wells containing P1 spheres 3 weeks later. Scale bar= 800 µm. **(c)** Flow cytometry plots show gating strategy for sorting transduced human $GFP^{+}CD298^{+}MHCI^{-}$ PDX cells for orthotopic transplantation and qPCR analysis. **(d)** Bar graph shows qPCR quantification of NME1 expression in control (+GFP) and NME1-GFP (+NME1) transduced cells. Values are plotted as fold change (FC) in NME1 expression in +NME1 relative to +GFP cells and represented as mean ± s.d. *P*-value determined by unpaired t-test.
