# Supplementary material for: Patient-derived xenograft culture-transplant system for investigation of human breast cancer metastasis
Source: Commun Biol. 2021 Nov 5;4:1268. doi: 10.1038/s42003-021-02596-y (PMC8571269; doi:10.1038/s42003-021-02596-y)
Supplement: Supplementary file 3 — Description of Supplementary Files [file 42003_2021_2596_MOESM3_ESM.pdf]

Description of Additional Supplementary Files

File name: Supplementary Movies 1-3

Description: Growth of HCI010 cells in MAT-E culture conditions. Time-lapse imaging of HCI010 PDX cells in MAT-E culture at day 7 post seeding. Growth shown over the course of 48 hours.

File name: Supplementary Data 1

Description: Identification of genes differentially in PDX cells after culture. Differential expression analysis identified 1,732 genes up and downregulated following MAT-E culture that were conserved across all PDX models (logFC>2.0, p<0.05). Adjusted P-values were determined in DESeq2 by Benjamini-Hochberg adjustment of Wald test P-values.

File name: Supplementary Data 2

Description: Source data for Figures 1-7.
