# Supplementary material for: Patient-derived xenograft culture-transplant system for investigation of human breast cancer metastasis
Source: Commun Biol. 2021 Nov 5;4:1268. doi: 10.1038/s42003-021-02596-y (PMC8571269; doi:10.1038/s42003-021-02596-y)
Supplement: Supplementary file 9 — Reporting Summary [file 42003_2021_2596_MOESM9_ESM.pdf]

nature research

Corresponding author(s): Devon A Lawson

Last updated by author(s): Feb 26, 2021

Reporting Summary

Nature Research wishes to improve the reproducibility of the work that we publish. This form provides structure for consistency and transparency in reporting. For further information on Nature Research policies, see our Editorial Policies and the Editorial Policy Checklist.

Statistics

For all statistical analyses, confirm that the following items are present in the figure legend, table legend, main text, or Methods section.

n/a	Confirmed	
☐	☒	The exact sample size (*n*) for each experimental group/condition, given as a discrete number and unit of measurement
☐	☒	A statement on whether measurements were taken from distinct samples or whether the same sample was measured repeatedly
☐	☒	The statistical test(s) used AND whether they are one- or two-sided<br>*Only common tests should be described solely by name; describe more complex techniques in the Methods section.*
☐	☒	A description of all covariates tested
☐	☒	A description of any assumptions or corrections, such as tests of normality and adjustment for multiple comparisons
☐	☒	A full description of the statistical parameters including central tendency (e.g. means) or other basic estimates (e.g. regression coefficient) AND variation (e.g. standard deviation) or associated estimates of uncertainty (e.g. confidence intervals)
☐	☒	For null hypothesis testing, the test statistic (e.g. *F*, *t*, *r*) with confidence intervals, effect sizes, degrees of freedom and *P* value noted<br>*Give P values as exact values whenever suitable.*
☒	☐	For Bayesian analysis, information on the choice of priors and Markov chain Monte Carlo settings
☒	☐	For hierarchical and complex designs, identification of the appropriate level for tests and full reporting of outcomes
☐	☒	Estimates of effect sizes (e.g. Cohen's *d*, Pearson's *r*), indicating how they were calculated

Our web collection on statistics for biologists contains articles on many of the points above.

Software and code

Policy information about availability of computer code

Data collection	Paired-end reads were aligned to reference genome GRCh38/hg38 from UCSC using Spliced Transcripts Alignment to a Reference (STAR) software. FACS data was collected in FACS diva software (version 6.1.1).
Data analysis	Data analysis for RNA-seq was performed in R (version 4.0.0). Differential expression analysis was performed using DESeq2 (version 1.28.1). Gene ontology analysis was done using Enrichr. Flow cytometry data analysis was performed using FlowJo (version 10.5.3).

For manuscripts utilizing custom algorithms or software that are central to the research but not yet described in published literature, software must be made available to editors and reviewers. We strongly encourage code deposition in a community repository (e.g. GitHub). See the Nature Research guidelines for submitting code & software for further information.

Data

Policy information about availability of data

All manuscripts must include a data availability statement. This statement should provide the following information, where applicable:

- Accession codes, unique identifiers, or web links for publicly available datasets
- A list of figures that have associated raw data
- A description of any restrictions on data availability

The authors declare that all data supporting the findings of this study are available within the article and its supplementary information files or from the corresponding author on reasonable request. All RNA-seq data files along with their associated metadata have been deposited in the GEO database under the accession code GSE153887.

April 2020

Field-specific reporting

Please select the one below that is the best fit for your research. If you are not sure, read the appropriate sections before making your selection.

- [x] Life sciences
- [ ] Behavioural & social sciences
- [ ] Ecological, evolutionary & environmental sciences

For a reference copy of the document with all sections, see nature.com/documents/nr-reporting-summary-flat.pdf

Life sciences study design

All studies must disclose on these points even when the disclosure is negative.

Sample size	Sample size for RNA sequencing was n=12. We ensured that each patient (HCI010 and HCI002) had 3 replicates for uncultured cell and cultured cell populations. We used triplicates for each sample and condition because this is standard for sequencing experiments.
Data exclusions	No data was excluded in the manuscript unless otherwise specified.
Replication	All attempts at replication were successful.
Randomization	We used mice for our model organism. Female mice were randomly assigned PDX tumours for transplant, or cultured PDX cells for injection.
Blinding	Blinding was not relevant to the study.

Reporting for specific materials, systems and methods

We require information from authors about some types of materials, experimental systems and methods used in many studies. Here, indicate whether each material, system or method listed is relevant to your study. If you are not sure if a list item applies to your research, read the appropriate section before selecting a response.

Materials & experimental systems

n/a	Involved in the study	
[ ]	[x]	Antibodies
[ ]	[x]	Eukaryotic cell lines
[x]	[ ]	Palaeontology and archaeology
[ ]	[x]	Animals and other organisms
[ ]	[x]	Human research participants
[x]	[ ]	Clinical data
[x]	[ ]	Dual use research of concern

Methods

n/a	Involved in the study	
[x]	[ ]	ChIP-seq
[ ]	[x]	Flow cytometry
[x]	[ ]	MRI-based neuroimaging

Antibodies

Antibodies used	CD298, diluted 1:100 (PE, BioLegend, Cat. No. 341704, Clone No. LNH-94, Lot No. B269993), MHC-I, diluted (APC, ThermoFisher, Scientific Cat. No. 17-5957-80, Clone No. SF1-1.1.1, Lot No. 2044729), aV-FITC, diluted 1:100 (GeneTex Cat. No. GTX14082), PI, diluted 1:100 (ThermoFisher Scientific Cat. No. P3566)
Validation	Human-specific CD298: The antibody was purified affinity chromatography and conjugated with PE. Each lot is quality control tested by immunofluorescent staining and flow cytometric analysis. For flow cytometry, the suggested use is 5ul per million cells in 100ul ul staining volume. References: Chiampanichayakul S, et al. 2006. Tissue Antigens; Malik N, et al. 1996. J. Biol. Chem.<br><br>Mouse-specific MHC-I: Antibody was validated by staining of C57BL/6 and BALB/c splenocytes. References: Kindy MS, et al. J. Transl. Med. 2016; Larsen J, et al. Eur. J. Immunol. 2014; Luo Y, et al. Stem Cells 2012; Horst D, et al. Cancer Res. 2012.<br><br>Annexin-V FITC: Annexin V has a strong, Ca2+-dependent affinity for PS and therefore is used as a probe for detecting apoptosis. Recommended use for FACS is 1 µl for 1 x 10^5 cells in 500 µl of 1X Annexin V Binding Buffer.<br><br>Propidium iodide (PI): PI is widely used and validated in fluorescence microscopy, confocal laser scanning microscopy, flow cytometry, and fluorometry. References: Medin, J A et al. 1996. Blood; Lackey, Chantal A et al. 2002. Bioconjug Chem.

Eukaryotic cell lines

Policy information about cell lines

Cell line source(s)	N/A

Authentication	N/A
Mycoplasma contamination	N/A
Commonly misidentified lines (See ICLAC register)	N/A

Animals and other organisms

Policy information about studies involving animals; ARRIVE guidelines recommended for reporting animal research

Laboratory animals	Orthotopic transplants of PDX tumours and cultured cell injections were done on 3-4 week old female NOD/SCID and NSG mice using established protocols.
Wild animals	The study did not include wild animals.
Field-collected samples	The study did not include field-collected samples.
Ethics oversight	The University of California, Irvine Institutional Animal Care and Use Committee (IACUC) reviewed and approved all animal experiments under AUP-19-051.

Note that full information on the approval of the study protocol must also be provided in the manuscript.

Human research participants

Policy information about studies involving human research participants

Population characteristics	Patient samples were generously provided by A.L. Welm in the Department of Oncological Sciences at the Huntsman Cancer Institute (HCI). HCI002 was acquired from a primary tumour biopsy of a female patient diagnosed with ER-PR-Her2-, basal-like Stage IIIA medullary type IDC with no previous systemic treatment. HCI010 was acquired from a pleural effusion of a Stage IIIC female patient diagnosed with ER-PR-Her2-, basal-like (PAM50) IDC treated with several rounds of chemotherapies.
Recruitment	All tissue samples were collected with informed consent from individuals being treated at the Huntsman Cancer Hospital and the University of Utah.
Ethics oversight	Samples were collected and de-identified by the Huntsman Cancer Institute Tissue Resource and Application Core facility before being obtained for implantation under a protocol approved by the University of Utah Institutional Review Board.

Note that full information on the approval of the study protocol must also be provided in the manuscript.

Flow Cytometry

Plots

Confirm that:

- [x] The axis labels state the marker and fluorochrome used (e.g. CD4-FITC).
- [x] The axis scales are clearly visible. Include numbers along axes only for bottom left plot of group (a 'group' is an analysis of identical markers).
- [x] All plots are contour plots with outliers or pseudocolor plots.
- [x] A numerical value for number of cells or percentage (with statistics) is provided.

Methodology

Sample preparation	After 3-6 months of growth, tumors were collected from mice and mechanically dissociated, followed by 2 mg/mL collagenase (Sigma-Aldrich, cat. no. C5138-1G) digestion in medium (DMEM F-12 medium with 5% FBS) at 37°C for 45 minutes on a standard shaker. The digested tumor was washed with PBS, incubated with trypsin (Corning, cat. no. 25-052-Cl) for 10 minutes at 37°C, washed again, and then treated with DNaseI (Worthington Biochemical, cat. no. LS002139). The cells were filtered through a 100 µm strainer. Live cell concentration was checked using the Countess II automated cell counter (ThermoFisher Scientific Inc., Carlsbad, CA, USA). We then cultured the PDX cells in a 1:1 mix of Matrigel (Corning, cat. no. 356231) and EpiCult Medium (StemCell Technologies, cat. no. 05610). After two weeks, we stained the cultured cells for FACS using fluorescent labeled antibodies for human specific CD298 (Biolegend, cat. no. 341704) and mouse-specific MHC-I (eBioscience, cat. no. 17-5957-80).
Instrument	BD FACSAria Fusion cell sorter
Software	FACS diva software (version 6.1.1), FlowJo (version 10.5.3)
Cell population abundance	CD298+ MHC-I- cells were sorted for downstream sequencing. After culturing cells, we commonly achieve a population of cells that is >85% CD298+ MHC-I-.
Gating strategy	Gating strategy first excludes doublet and multiplet cells by forward scatter area x forward scatter width (FSC W x FSC A) and

side scatter area x side scatter width (SSC W x SSC A). We then gate on live cells, as determined by negative staining for SYTOX Blue (ThermoFisher Scientific Cat. No. S34857). Then we separate mouse and human cells by CD298 and MHC-1. Finally, we sort cells that are Sytox-CD298+MHC-I-.

☒ Tick this box to confirm that a figure exemplifying the gating strategy is provided in the Supplementary Information.
